# Comparative Analysis of Hydrodynamic Performance for Flapping Hydrofoils Driven by Three Typical Transmission Mechanisms

**DOI:** 10.3390/biomimetics10080549

**Published:** 2025-08-21

**Authors:** Ertian Hua, Sihan Li, Xiaopeng Wu, Yang Lin

**Affiliations:** College of Mechanical Engineering, Zhejiang University of Technology, Hangzhou 310023, China; sihli.work@gmail.com (S.L.); 211123020069@zjut.edu.cn (X.W.); 221123020245@zjut.edu.cn (Y.L.)

**Keywords:** bionic hydrofoil, numerical simulation, hydrodynamic performance, plains river network, motion mode, critical frequency

## Abstract

This study aims to optimize bionic hydrofoil propulsion performance and establish design guidelines for efficient transmission mechanisms by comparing three mechanisms (crank-slider, cylindrical cam, and synchronous belt drive). Through 3D modeling, virtual assembly, and ADAMS simulations, dynamic responses of slider displacement and driving force/torque were obtained, revealing that the crank-slider consumes the least energy, followed by the cylindrical cam, with the synchronous belt being the most energy-intensive. Further CFD analysis demonstrated that while the crank-slider generates drag intermittently, the cylindrical cam and synchronous belt sustain continuous thrust. All mechanisms achieve effective water propulsion below their critical frequencies (0.25 Hz, 0.75 Hz, and 1.4 Hz, respectively). Propulsion efficiency peaks at 26.0% (crank-slider) and 24.7% (cylindrical cam) at 0.25 Hz but declines at higher frequencies, whereas the synchronous belt reaches 24.3% efficiency at 1 Hz with superior frequency adaptability. The synchronous belt emerges as the optimal solution for efficient flapping propulsion due to its motion continuity and frequency adaptability. This work elucidates the critical impact of transmission mechanisms on hydrofoil hydrodynamics, providing foundational insights for mechanism design and performance optimization.

## 1. Introduction

To improve water mobility in main river channels, integrated operation of pumping stations and sluice gates is commonly adopted [1,2]. However, conventional pumps exhibit low efficiency under ultra-low head conditions, accompanied by severe vibration, noise generation, and cavitation issues [3,4]. To address these challenges, the flapping hydrofoil based on a fishtail oscillation has emerged as an innovative solution [5]. This study proposes three transmission mechanisms for flapping hydrofoils to enhance hydrodynamic performance under low-head operating conditions, with a comparative analysis of key performance parameters across different transmission configurations.

Bionic hydrofoils derive their design inspiration from fish locomotion. As demonstrated in Refs. [6,7,8], the caudal fin serves as the primary thrust generation mechanism in fish, with its hydrodynamic performance critically influencing propulsion efficiency. Regarding hydrofoil propulsion characteristics, Koochesfahani et al. revealed that oscillating frequency governs the shedding patterns of leading-edge vortices (LEVs) and trailing vortices, thereby determining thrust output [9]. Through experimental validation, Kármán et confirmed that the “jet flow” phenomenon in reverse Kármán vortex streets directly correlates with thrust generation, establishing the feasibility of flapping hydrofoils for aquatic propulsion [10].

Subsequent investigations refined this space by exploring the influence of foil geometry and motion parameters [11,12,13,14,15], tandem arrangements [16,17], non-sinusoidal waveforms [18,19] and energy harvesting [20], and even extended the concept to aquaculture raceways [21]. However, all of these investigations assumed perfectly sinusoidal motion imposed by ideal actuators. Only a handful of papers have addressed physical drive trains: five-bar linkages [22], serial spherical hinges [23], lead screw-gear parallel mechanisms [24], crank-rockers [25], spatial parallel joints [26], and servo-driven linkages [27]. Each introducing its own amplitude attenuation, phase lag, higher-harmonic content and frequency-dependent errors. No previous work has quantitatively benchmarked these transmission-induced kinematic deviations against the target motion, nor evaluated how the resulting loss of motion fidelity propagates into thrust generation and energy expenditure.

In summary, bionic hydrofoils have been extensively studied for underwater propulsion. However, practical applications in small river channels under ultra-low head and high-flow conditions remain underexplored, particularly regarding the design of hydrodynamic devices capable of providing optimal flow dynamics. Unlike our previous studies, which focused on hydrofoil geometry or tandem-phase effects under idealized kinematics [28,29], this work is the first to quantify how real transmission mechanisms (with their inherent motion distortions and power losses) dictate the operable frequency envelope and energy effectiveness of flapping hydrofoil devices. This work aims to provide critical insights for designing low-head-adapted flapping hydrofoil systems tailored to small river channel environments.

## 2. Structural Design of Flapping Hydrofoil Mechanisms

### 2.1. Mechanism Design of Flapping Hydrofoil Devices

**Figure 1:**
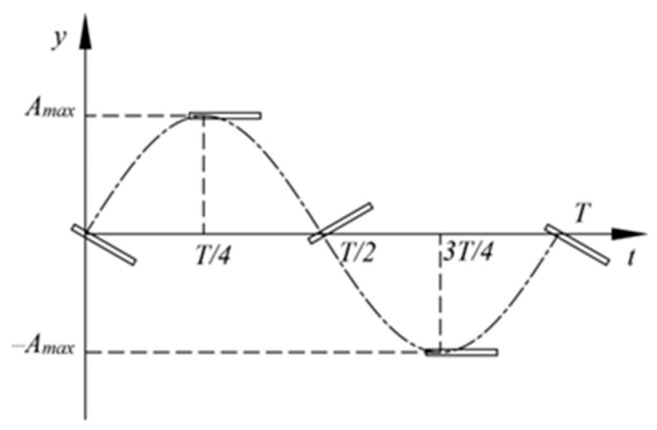
Schematic of hydrofoil heave and pitch motions.

The flapping hydrofoil structure adopts a flat-plate airfoil with a chord length C = 1 m. As shown in [28], positioning the pivot at 0.2C optimizes hydrodynamic performance. The flapping kinematics employs a combined heave-pitch motion pattern (as shown in Figure 1), with the fundamental kinematic equations expressed as follows:

(1) $$\left\{\begin{array}{c} y\left(t\right)=A_{\max}\sin\left(2\pi ft\right)\\ \theta \left(t\right)=\theta_{\max}\sin\left(2\pi ft+\varphi \right) \end{array}\right.,$$

where *y*(*t*) represents the heave displacement of the hydrofoil, *θ*(*t*) denotes the pitch angle of the hydrofoil, *f* is the combined motion frequency of the hydrofoil, and *φ* represents the phase difference between the heave and pitch motions of the hydrofoil, which is set to −*π*/2.

**Figure 2:**
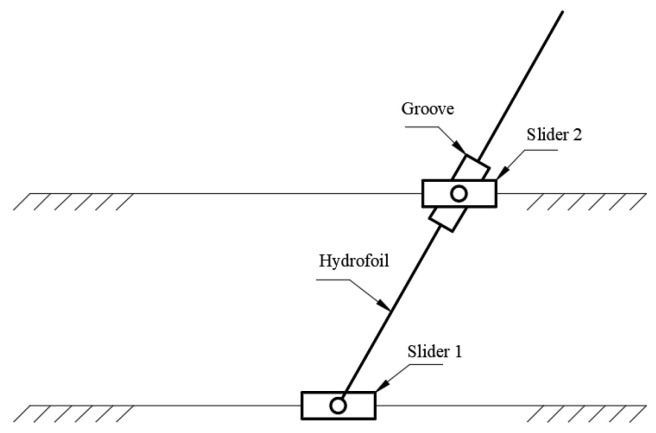
Hydrofoil motion implementation mechanism.

Figure 2 depicts the kinematic realization mechanism of the hydrofoil. The propulsion process is achieved by controlling two sliders: Slider 1, positioned at the hydrofoil’s pivot location (0.2C), drives the fixed rod to govern heave motion, while Slider 2, sliding within a groove on the hydrofoil connector, coordinates the pitch motion through rod actuation. This dual-slider mechanism enables synchronized heave-pitch oscillations, thereby generating controlled hydrodynamic thrust.

All three flapping hydrofoil mechanisms are engineered based on the prescribed kinematic principles. Mode 1 employs a crank-slider mechanism, where a servo motor synchronously rotates dual cranks via couplings, converting rotational motion into linear reciprocation to drive the hydrofoil. Mode 2 utilizes a cylindrical cam that governs the motion of a slider-linked rod through its rotational profile, enforcing predefined trajectory constraints on the slider. Mode 3 adopts a timing belt transmission system, with a microcontroller-programmed stepper motor driving the slider assembly to achieve precision motion control. These mechanisms are hereafter referred to as mode 1, mode 2, and mode 3, respectively.

### 2.2. Motion Simulation of Flapping Hydrofoil Device

**Figure 3:**
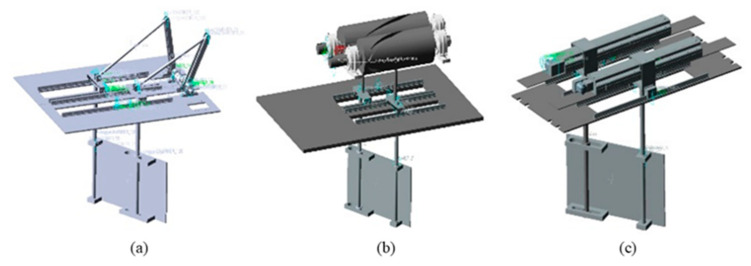
Virtual prototype modes of three configurations: (**a**) Mode 1; (**b**) Mode2; (**c**) Mode 3.

To simulate the kinematic posture of the flapping hydrofoil mechanism and validate the rationality of its structural motion, a comprehensive dynamic simulation was performed in ADAMS. The workflow proceeded as follows: The assembled 3D model of the mechanism was imported into the ADAMS/View environment, with material properties assigned to components—stainless steel for guide rails and sliders to meet corrosion resistance requirements, and aluminum alloy for other parts to reduce inertia. Motion constraints were then applied to construct the kinematic model, followed by the definition of driving forces/torques. As shown in Figure 3, the ADAMS/Solver was subsequently executed to perform kinematic-dynamic co-simulation, enabling rigorous analysis of the mechanism’s motion characteristics and energy transmission efficiency.

Due to the periodic nature of the flapping hydrofoil motion, modes 1 and 2 (both motor-driven) complete one full motion cycle per rotation of the crank-slider mechanism or cylindrical cam. The motor rotational speed was set to π/2 rad/s (equivalent to 90°/s), with a simulation duration of 8 s and 4000 time steps to ensure high temporal resolution. For Mode 3, a prismatic joint constraining the slider-rail assembly was programmed to follow a sinusoidal motion law, ensuring precise synchronization with the desired flapping kinematics.

**Figure 4:**
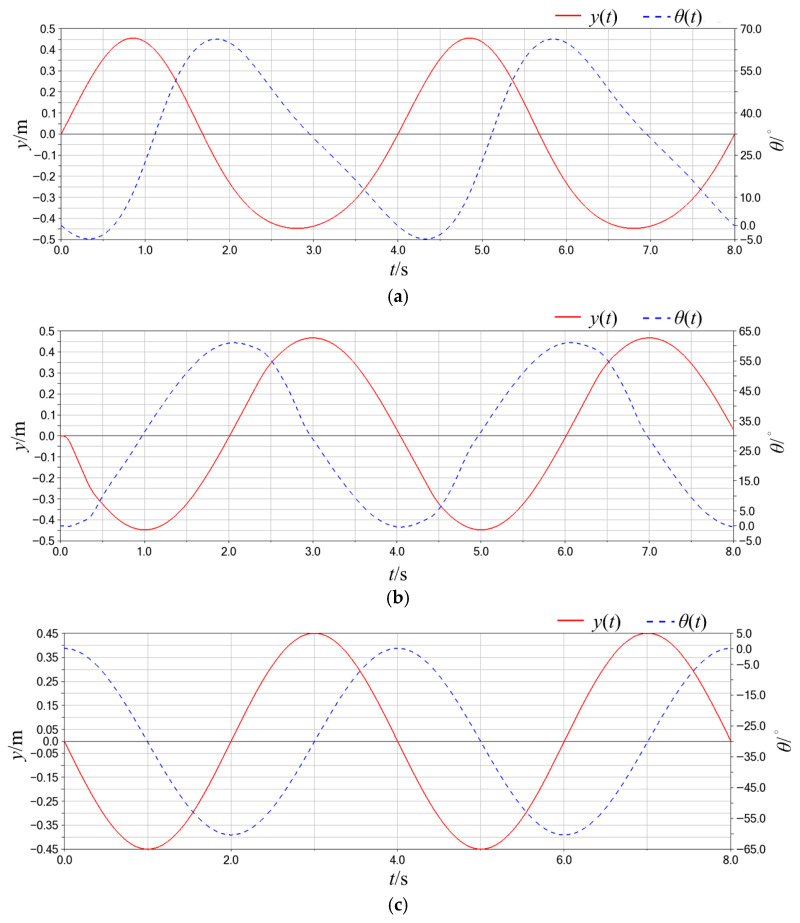
Hydrofoil heave and pitch displacement profiles: (**a**) Mode 1; (**b**) Mode 2; (**c**) Mode 3.

The simulation results, shown in Figure 4, present the heave and pitch displacement curves of the hydrofoil pivot over two motion cycles. Mode 1 exhibits velocity asymmetry due to the inherent quick-return characteristics of the mechanism, causing both heave and pitch motions to deviate from standard sinusoidal profiles, with an initial reverse-direction oscillation observed during startup. Mode 2 achieves a near-ideal sinusoidal heave trajectory but introduces asymmetric deviations in pitch motion due to cam profile tolerances. In contrast, Mode 3 demonstrates strict adherence to sinusoidal laws in both heave and pitch displacements, fulfilling the kinematic requirements with high fidelity.

### 2.3. Power Consumption Analysis of the Mechanisms

**Figure 5:**
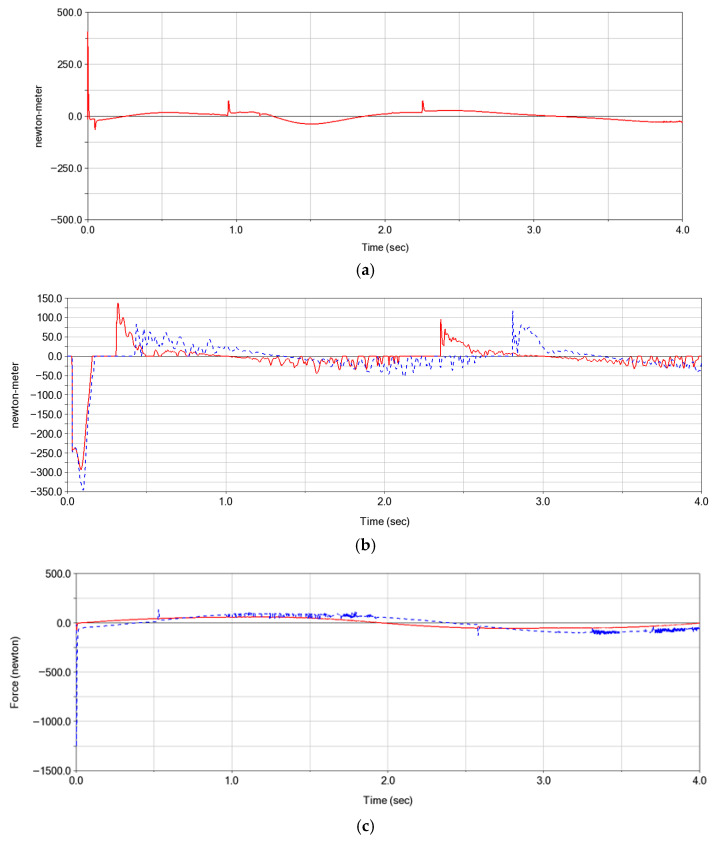
Actuation force and torque profiles of three mechanisms (**a**) Mode 1; (**b**) Mode 2; (**c**) Mode 3.

To minimize energy consumption while ensuring stable operation of the mechanisms, motor selection requires calculating the maximum driving torque (for rotary-driven modes 1 and 2) and driving force (for linear-driven mode 3). Figure 5 illustrates the dynamic driving torque/force profiles of the three mechanisms.

Based on the figure, Mode 1 exhibits an initial transient torque peak approaching 500 N·m at its starting position. This results from static friction inertia that must be overcome at the mechanism’s dead center position. After brief fluctuations, the system stabilizes with periodic torque oscillations, demonstrating motion stability enabled by its self-locking mechanism.

For Mode 2, the overall driving torque remains higher, peaking near 300 N·m. During operation, multiple torque discontinuities occur, correlating with contact force jumps induced by abrupt curvature changes in the cam profile. These irregularities lead to compromised motion smoothness.

Mode 3 requires overcoming system static friction during startup, generating a peak force of approximately 1500 N. Upon reaching steady-state operation, its driving force exhibits minimal fluctuations (±100 N). This behavior highlights the damping effect of belt transmission’s elasticity characteristics on shock loads.

Under the specified operating conditions, the maximum instantaneous power is calculated based on the time-series data of driving torque *T*(*t*)/force *F*(*t*) and corresponding angular velocity *ω*(*t*)/linear velocity *v*(*t*) output from ADAMS simulations.

(2) P(t)=T(t)·ω,

(3) P(t)=F(t)·v,

The maximum power consumption values for the three motion modes are approximately 784 W, 471 W, and 1177 W, respectively. Thus, under identical motion frequencies, Mode 1 demonstrates the lowest energy consumption, Mode 2 exhibits moderate energy expenditure, and Mode 3 requires the highest energy input.

## 3. Motion Mode

### 3.1. Motion Description

As described in preceding sections, the kinematic patterns of the three motion modes have been defined. To accurately compare their distinct effects, Fourier series analysis is adopted as the fitting model due to its mathematical rigor in representing periodic functions. Fourier series decomposes complex periodic waveforms into linear combinations of sine and cosine functions, expressed as:

(4) $$f(t)=a_{0}+\sum_{n=1}^{N}\left(a_{n}\cos(n\omega t)+b_{n}\sin(n\omega t)\right),$$

In the equation, *a*_0_ represents the constant term, *a_n_* and *b_n_* denote the cosine and sine coefficients of each harmonic component, *ω* is the fundamental frequency, and *N* indicates the selected order of the Fourier series. The motion curves of Modes 1 and 2 were imported into MATLAB-R2022a for processing. After fitting, the resulting Fourier series models were applied to subsequent calculations.

### 3.2. Performance Parameters

In this study, a rigid flat-plate hydrofoil is analyzed with prescribed kinematic parameters: heave amplitude *H*_max_ = 0.45 m and pitch amplitude *θ*_max_ = 30°. During hydrofoil motion, the Strouhal number (*St*), a key dimensionless parameter characterizing the wake flow dynamics, is defined as

(5) $$St=\frac{2fH_{\max}}{U},$$

where *U* is free-stream velocity.

To facilitate the research, the thrust and lift forces generated during the oscillatory motion of the hydrofoil are non-dimensionalized to obtain the instantaneous thrust coefficient:

(6) $$C_{T}=\frac{2F_{x}(t)}{\rho U^{2}cs},$$

Instantaneous lift coefficient:

(7) $$C_{L}=\frac{2F_{y}(t)}{\rho U^{2}cs}\;,$$

where *F_x_*(*t*) is the instantaneous thrust; *F_y_*(*t*) is instantaneous lift; *ρ* is the fluid density; *c* is the chord length of the airfoil; *s* is the span length of the airfoil.

The mean thrust coefficient and lift coefficient over several complete operating cycles:

(8) $$\bar{C_{T}}=\frac{1}{KT}\int_{(n-k)T}^{nT}C_{T}dt\;,$$

(9) $$\bar{C_{L}}=\frac{1}{KT}\int_{(n-k)T}^{nT}C_{L}dt\;,$$

where *K* is the integer number of computational cycles, and this paper sets *K* = 2.

To further investigate the propulsion performance of the flapping hydrofoil, the following performance parameters are introduced:

The outlet average flow rate of a steady flow field is defined as

(10) $$\bar{Q}=US,$$

where *S* is the cross-sectional area of the flow passage.

Average input power is defined as

(11) $$\bar{P}=\frac{1}{T}\left(\int_{0}^{T}F_{y}(t)\dot{y}(t)dt+\int_{0}^{T}M(t)\dot{\theta }(t)dt\right),$$

where *M*(*t*) is the instantaneous driving torque.

Mean power coefficient is defined as

(12) $$\bar{C_{p}}=\frac{2\bar{P}}{\rho csU^{3}},$$

The propulsion efficiency of the flapping hydrofoil is defined as

(13) $$\eta =\frac{\bar{C_{T}}}{\bar{C_{p}}},$$

The average power harvested by the fluid is defined as

(14) $$\bar{P_{w}}=\Delta \bar{P}\cdot \bar{Q},$$

where ∆$\bar{P}$ is the average pressure difference between the inlet and outlet.

The average head is defined as

(15) $$\bar{H}=\frac{\Delta \bar{P}}{\rho g},$$

The pumping efficiency of the system is defined as

(16) $$\eta_{p}=\frac{\bar{P_{w}}}{\bar{P}},$$

### 3.3. Governing Equations and Turbulence Model

This study employs the commercial CFD software ANSYS FLUENT-2024R2 for numerical simulations. The governing equations include the Reynolds-Averaged Navier-Stokes (RANS) equations for viscous, two-dimensional, incompressible flow, along with the time-averaged continuity equation. The motion control equations are [30]:

(17) $$\frac{\partial {\bar{u}}_{i}}{\partial x_{i}}=0,$$

(18) $$\frac{\partial {\bar{u}}_{i}}{\partial t}+{\bar{u}}_{j}\frac{\partial {\bar{u}}_{i}}{\partial x_{j}}=-\frac{\partial \bar{p}}{\partial x_{i}}+\frac{\partial }{\partial x_{j}}\left[(\gamma +\gamma_{i})\left(\frac{\partial {\bar{u}}_{i}}{\partial x_{j}}+\frac{\partial {\bar{u}}_{j}}{\partial x_{i}}\right)\right],$$

where $\bar{u}$*_i_* (*i* = 1, 2) is the mean velocity of fluid motion, *x_i_* (*i* = 1, 2) is the spatial coordinates, *p* is the fluid pressure, *t* is the time, *γ* is the kinematic viscosity coefficient, *γ_i_* = *c_μ_k*^2^/*ε* is the turbulent viscosity coefficient, where *c_μ_* is a model constant, *k* is the turbulent kinetic energy, *ε* is the turbulent kinetic energy dissipation rate.

During the oscillatory motion of the hydrofoil, vortex generation and shedding directly influence the propulsion performance. To accurately capture near-wall flow characteristics and align the model with the research objectives, the Realizable *k*-*ε* turbulence model is adopted for solving the Navier-Stokes equations.

The most prevalent *k*-epsilon (*k-ε*) and *k*-omega (*k-ω*) turbulence models in Fluent both derive from formulations of turbulent kinetic energy and turbulent dissipation rate, yet the *k-ω* model suffers from inferior convergence and extended computational demands. Consequently, this study employs the computationally superior *k-ε* framework, which encompasses three variants: Standard, RNG, and Realizable. While the Standard *k-ε* model delivers high efficiency suitable for broad engineering applications, it demonstrates inherent limitations in resolving near-wall boundary layers. The RNG *k-ε* variant enhances wall-effect predictions through mathematical refinements at the cost of elevated computational overhead, whereas the Realizable *k-ε* model—by integrating statistical correlations of turbulent stresses—achieves superior accuracy and reliability in capturing turbulent anisotropy and near-wall physics, rendering it optimally suited for the hydrofoil vortex shedding phenomena central to this investigation. The specific governing equations for this model are detailed in [30].

### 3.4. Verification of the Independence of the Grid Number and Time Steps

**Figure 6:**
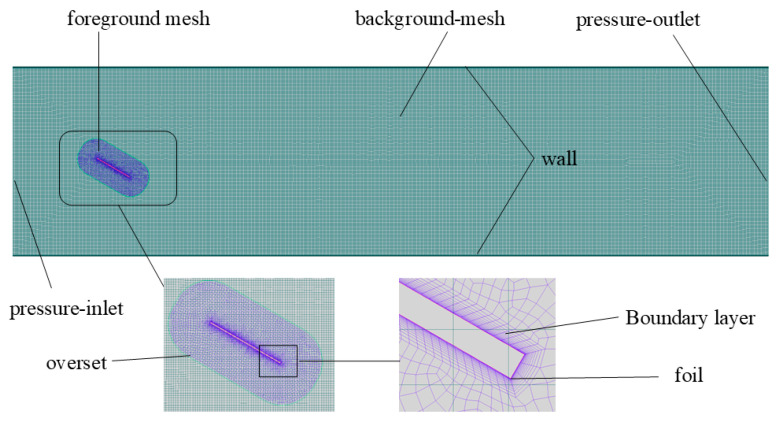
Mesh generation schematic.

Considering the application of the flapping hydrofoil system in urban waterways within river network regions, the fluid domain dimensions are set to 20c × 5c (where c is the chord length). To effectively capture trailing vortices and avoid mesh negative volume issues, this study employs User-Defined Functions (UDFs) combined with overset grid technology to handle hydrofoil motion. The computational domain comprises a foreground grid and a background grid, as shown in Figure 6. The foreground grid represents the flapping hydrofoil motion, whose kinematic parameters are governed by UDFs, while the background grid corresponds to a rectangular water channel. Boundary conditions are specified as follows: pressure-inlet at the fluid domain entrance, pressure-outlet at the exit, overset boundary for the outermost interface of the foreground grid, and no-slip walls for both the hydrofoil surface and channel sidewalls.

Different turbulence models and wall functions impose distinct requirements on the grid y+ values. To more accurately capture near-wall flow characteristics, boundary layer grids were implemented on both the hydrofoil surface and flow channel walls, employing an enhanced wall treatment approach. This method achieves refined resolution of the viscous sublayer by positioning the first grid layer within the viscous sublayer. The initial boundary layer height was set to 0.0464 mm with a grid growth ratio of 1.2, ensuring y+ values < 1 throughout simulations. Numerical solutions are obtained using FLUENT’s pressure-based solver to resolve the Navier-Stokes equations. The Coupled scheme is implemented for pressure-velocity coupling, with First-Order Upwind discretization applied to the turbulent kinetic energy and dissipation rate equations, and Second-Order Upwind discretization adopted for the momentum equations.

### 3.5. Grid Independence Verification

**Figure 7:**
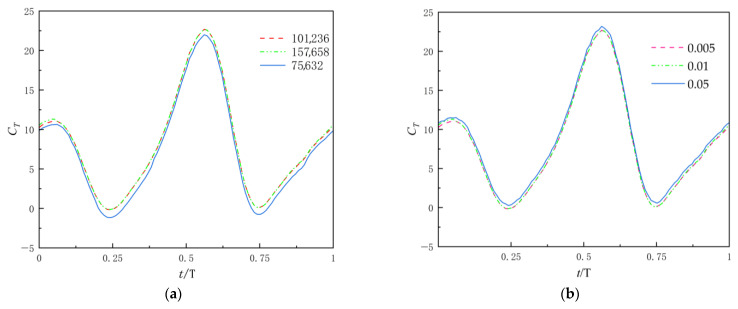
Verification of independence: (**a**) grid number; (**b**) time steps.

To ensure computational accuracy and improve simulation efficiency, a grid independence study was conducted by evaluating three mesh configurations with grid counts of 75,632, 101,236, and 157,658 under Mode 3 of harmonic motion. As shown in Figure 7a. By comparing the instantaneous thrust coefficient variation curves of the flapping hydrofoil across these meshes, significant errors were observed for the 75,632-cell mesh. However, negligible discrepancies were found between the 101,236-cell and 157,658-cell meshes, indicating that increasing the grid count beyond 101,236 cells had no appreciable impact on the results. Thus, the 101,236-cell mesh was selected as the optimal configuration.

To verify temporal independence, we conducted numerical simulations using three distinct time step sizes—0.05 s, 0.01 s, and 0.005 s—with comparative results presented in Figure 7b. This analysis revealed that a time step of 0.05 s produced significant deviations in instantaneous thrust coefficient, whereas the 0.01 s and 0.005 s steps exhibited markedly smaller discrepancies. Consequently, balancing computational accuracy with efficiency, we selected 0.01 s as the optimal time step for all subsequent simulations.

### 3.6. Validation of Numerical Methodology

**Figure 8:**
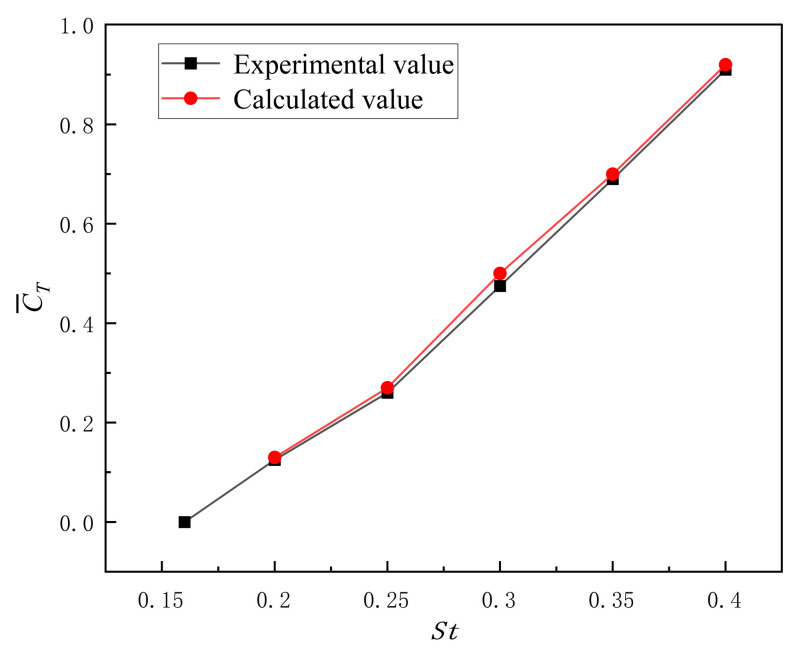
Numerical simulated thrust compared with experimental data from literature.

To verify the feasibility of the numerical methodology employed in this study, the NACA 0012 hydrofoil was selected for validation by comparing computational results with experimental data from Reference [31]. The simulation parameters strictly replicated the experimental conditions documented in the reference, including the inlet velocity *U* = 0.4 m/s, maximum angle of attack *θ*_max_ = 30°, phase angle *φ* = −π/2, and heave amplitude A_max_ = C = 0.1 m. The computed average thrust coefficient versus Strouhal number curve for the flapping hydrofoil was systematically compared with the reference data. The numerical results demonstrate close agreement with experimental measurements, exhibiting excellent consistency in both trend and magnitude. This rigorous validation confirms the reliability and accuracy of the present computational approach in resolving unsteady hydrodynamic phenomena. The results are shown in Figure 8.

## 4. Analysis of Computational Results

To investigate the propulsion mechanisms and performance of the hydrofoil under different driving mechanisms, the flapping frequency *f* of the hydrofoil was adjusted by varying the operating speeds of three driving mechanisms. The frequency range was set to *f* = 0.25–2.0 Hz, with increments of 0.25 Hz in the 0.25–1.0 Hz sub-range and 0.2 Hz in the 1.0–2.0 Hz sub-range. Data from the last two flapping cycles after achieving computational stability were selected for analysis.

### 4.1. Influence of Motion Modes on Thrust and Lift

**Figure 9:**
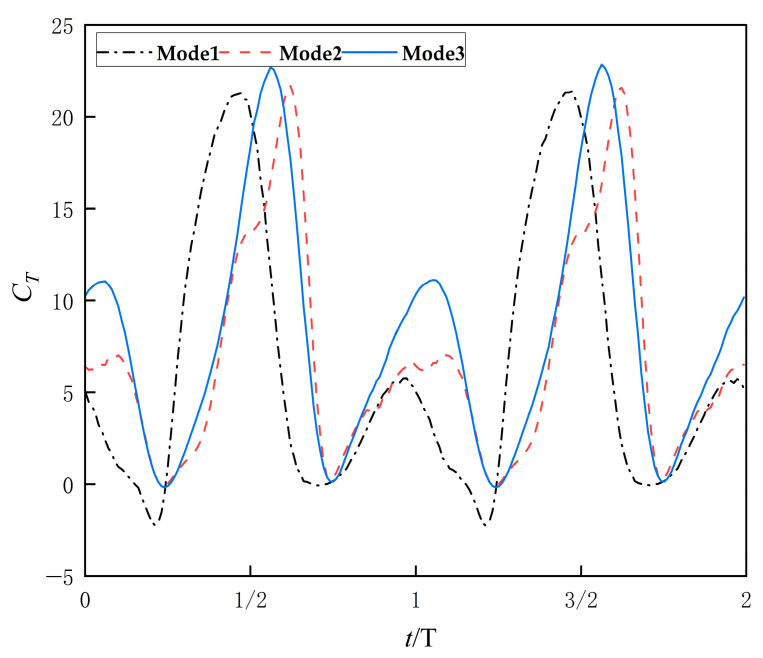
Instantaneous thrust coefficient profiles of three flapping modes.

Figure 9 shows the time-dependent thrust coefficient (*C_T_*) curves for different motion patterns over two flapping cycles. As shown in the figure, the instantaneous thrust coefficient (*C_T_*) of the hydrofoil exhibits distinct periodic variations, with a period identical to the flapping motion cycle of the hydrofoil. Each cycle contains two pronounced peaks and two troughs in *C_T_*. While the three motion modes share consistent trends in *C_T_* variation, the following phase-specific behaviors are observed: Starting from the equilibrium position, *C_T_* rapidly decreases to a trough before the hydrofoil reaches its maximum flapping amplitude. Subsequently, *C_T_* gradually rises, peaking just before the hydrofoil returns to the equilibrium position. As the hydrofoil swings in the reverse direction, *C_T_* declines again, reaching another trough near the maximum reverse flapping amplitude. Finally, *C_T_* peaks once more as the hydrofoil approaches the equilibrium position.

Among the three motion methods, Mode 1 exhibits higher peak values in the instantaneous thrust coefficient (*C_T_*) but significant negative thrust (resistance), indicating energy loss due to drag during motion. Analysis of the figure reveals that during the initial motion phase, the hydrofoil undergoes a reverse swing, reaching a stall angle of attack (>30°), which triggers a sudden surge in pressure drag. In contrast, Mode 2 demonstrates smoother Ct fluctuations with smaller amplitude, and *C_T_* remains positive throughout the cycle, ensuring continuous thrust generation. Mode 3 achieves the highest peak *C_T_* values while maintaining *C_T_* > 0, with even gentler variations compared to Mode 2, suggesting superior thrust stability.

**Figure 10:**
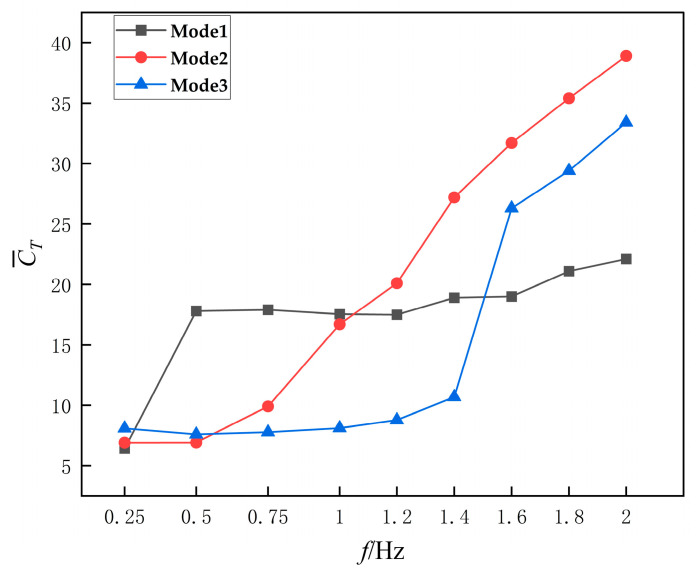
Average thrust coefficient profiles of three flapping modes.

To investigate the thrust variation of the three motion modes under different frequencies, the thrust values obtained from numerical simulations were non-dimensionalized, and the mean thrust coefficient of the flapping hydrofoil was plotted against frequency, as shown in Figure 10. The results indicate that the mean thrust coefficients for all three motion modes increase with frequency and remain positive, demonstrating that all three driving mechanisms effectively propel the fluid within their respective frequency ranges. Specifically: Mode 1 shows a relatively smaller increase in thrust coefficient with frequency. Mode 2 exhibits a faster increase rate in thrust coefficient. Mode 3 maintains a higher thrust coefficient value despite a more gradual increase compared to Mode 2.

**Figure 11:**
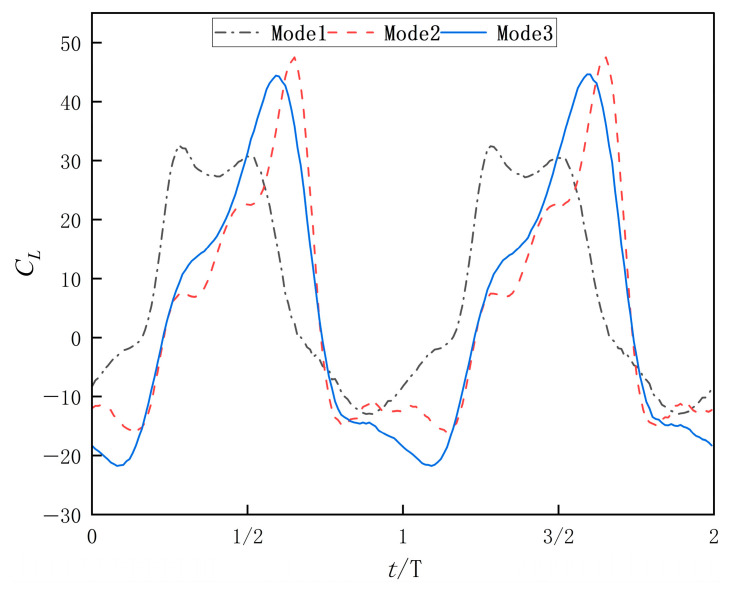
Instantaneous lift coefficient profiles of three flapping modes.

Figure 11 displays the time-dependent instantaneous lift coefficient (*C_L_*) curves over two flapping cycles. As shown, the instantaneous lift coefficient also exhibits periodic behavior: Mode 1 achieves higher lift peaks but displays complex fluctuations with multiple local peaks, indicating unstable lift generation. Mode 2 attains the highest lift peaks, concentrated in distinct phases, and demonstrates smoother fluctuations, suggesting strong and stable lift output. Mode 3 shows slightly lower lift peaks than Mode 2, but its curve is significantly smoother, with more uniform lift growth and decay phases and smaller fluctuation amplitudes.

### 4.2. Influence of Motion Modes on Wake Vortex Structures

**Figure 12:**
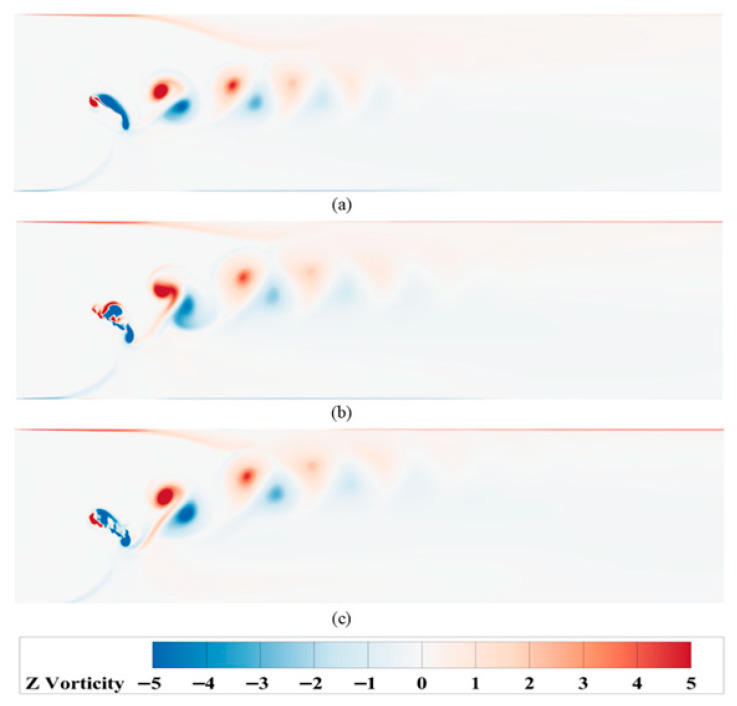
Schematic representations of vortex structures for hydrofoils under different motion modes (**a**) Mode 1; (**b**) Mode 2; (**c**) Mode 3.

The periodic variations in instantaneous thrust and lift coefficients, as analyzed earlier, suggest a need to investigate the underlying fluid dynamic mechanisms. To this end, the wake vortex structures generated during the hydrofoil’s oscillation were analyzed. Figure 12 shows the vorticity contours of the wake vortices for the three motion methods at a frequency of *f* = 0.25 Hz.

**Figure 13:**
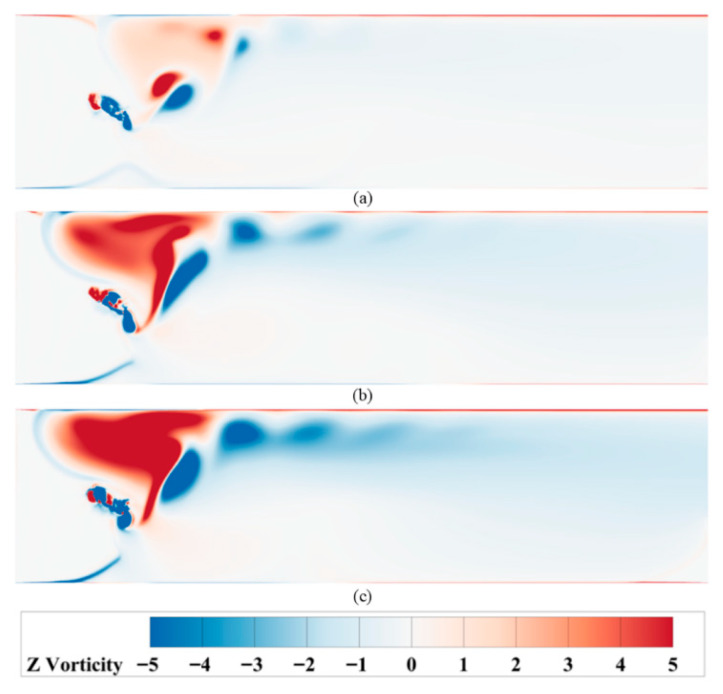
Vortex structures of three motion modes at different frequencies (**a**) Mode 1: *f* > 0.25 Hz; (**b**) Mode 2: *f* > 0.75 Hz; (**c**) Mode 3: *f* > 1.4 Hz.

As shown in the analysis, under low-frequency conditions, all three motion modes generate stable reverse von Kármán vortex streets around the hydrofoil, with alternating negative vortices (clockwise) and positive vortices (counterclockwise) forming a regular structure. This vortex shedding pattern significantly enhances flow field stability and thrust efficiency. However, as the motion frequency increases, vortex formation becomes unstable: negative vortices rapidly generate and detach near the hydrofoil surface, accompanied by pronounced asymmetry in vorticity distribution. Specifically, Mode 1 exhibits severe vortex street offset due to its quick-return characteristic, where the asymmetric heaving velocities intensify with frequency, causing negative vortices to accumulate at the wall boundary and induce backflow. Mode 2 can maintain a vortex street at the motion frequency *f* = 0.75 Hz. However, when the frequency exceeds 0.75 Hz, significant flow reversal occurs. This phenomenon is attributed to the enhanced symmetry of Mode 2’s kinematics compared to Mode 1, which expands its frequency range for sustaining stable vortex street formation. In contrast, Mode 3 exhibits discernible vortex street deflection only when the motion frequency exceeds 1.4 Hz. This phenomenon originates from the hydrodynamic interaction during the hydrofoil’s startup phase in quiescent water: As the hydrofoil initiates upward motion, it imparts an upward force to the surrounding flow field, generating an initial upward velocity component in the fluid domain. This residual velocity field interacts with high-frequency oscillations, resulting in systematic vortex pattern deviation, as shown in Figure 13.

Therefore, under high-frequency conditions, intensified flow disturbances and vorticity disrupt the vortex structure. This suggests that high-frequency motion may induce stronger vortices and instabilities, compromising the hydrofoil’s thrust efficiency. Among the three mode, Mode1 exhibits the broadest operable frequency range with optimal propulsion performance, followed by Mode 2, while Mode 1 has the narrowest applicable frequency range due to its inherent instability.

### 4.3. Analysis of Motion Mode Effects on Hydrodynamic Propulsion

**Figure 14:**
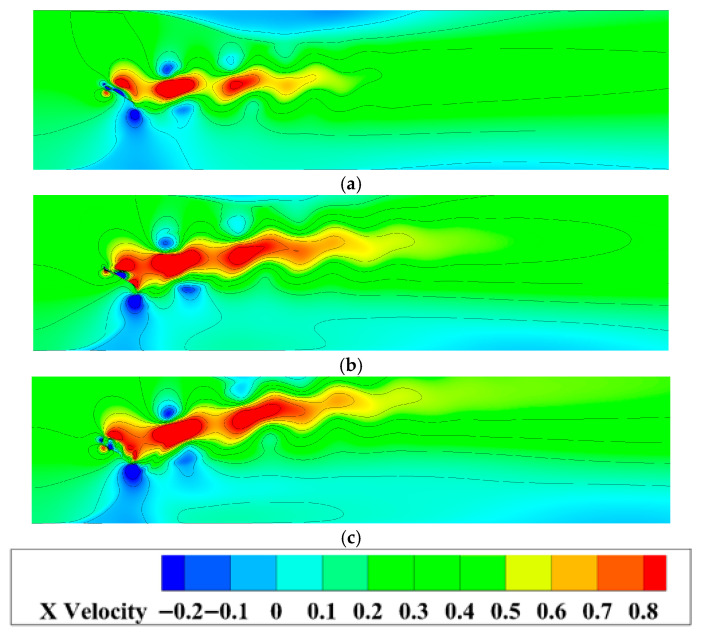
Velocity contours of the hydrofoil under different motion modes at *f* = 0.25 Hz: (**a**) Mode 1; (**b**) Mode 2 (**c**) Mode 3.

Due to their distinct kinematic characteristics, the three flapping hydrofoil configurations exhibit significantly different propulsion performances. A comparative analysis of their velocity contour maps at *f* = 0.25 Hz is shown in Figure 14.

**Figure 15:**
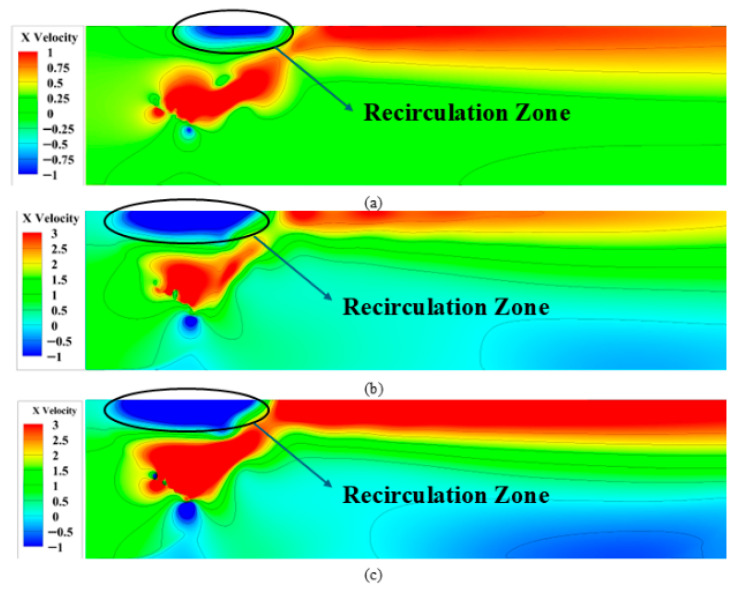
Velocity contours of three motion modes at different frequencies (**a**) Mode 1: *f* > 0.25 Hz; (**b**) Mode 2: *f* > 0.75 Hz; (**c**) Mode 3: *f* > 1.4 Hz.

Observations indicate that all three motion modes generate stable jet flows, with velocity magnitudes exhibiting radial gradients from the central region toward both sides. Specifically, Mode 1 demonstrates the most stable jet profile, while Mode 2 and Mode 3 exhibit upward deflection in their jet streams. Analysed in conjunction with Figure 12, this upward jet bias in Mode 2 and 3 directly correlates with the upward offset of their trailing vortices at this frequency, thereby inducing flow asymmetry. However, no significant backflow is observed under this low-frequency condition. In contrast, as the frequency increases, all three modes develop pronounced backflow at their respective critical frequencies, as shown in Figure 15.

**Figure 16:**
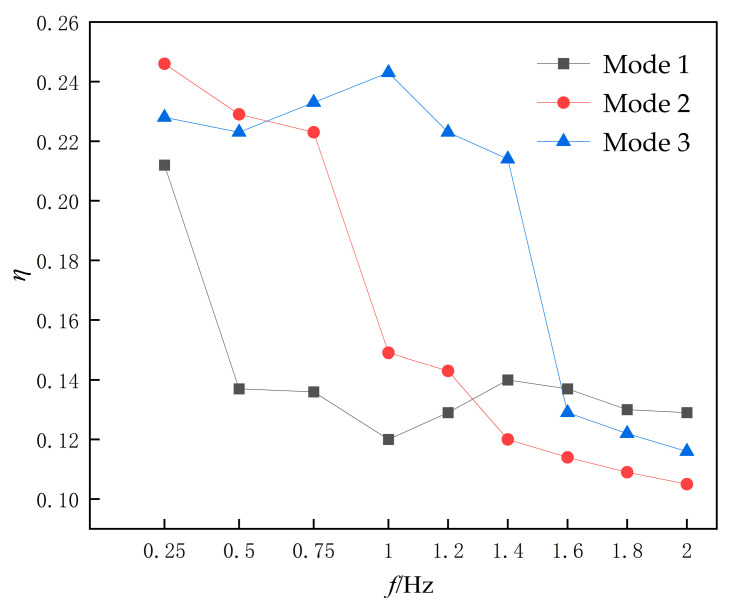
Propulsive efficiency profiles of different flapping modes as a function of flapping frequency.

To further investigate the impact of different flapping methods on the water-pushing performance of hydrofoils, variation curves of propulsion efficiency and pumping efficiency of the hydrofoil with respect to oscillation frequency were plotted. As shown in Figure 16, the propulsion efficiency curve of the flapping hydrofoil device demonstrates its dependence on flapping frequency.

**Figure 17:**
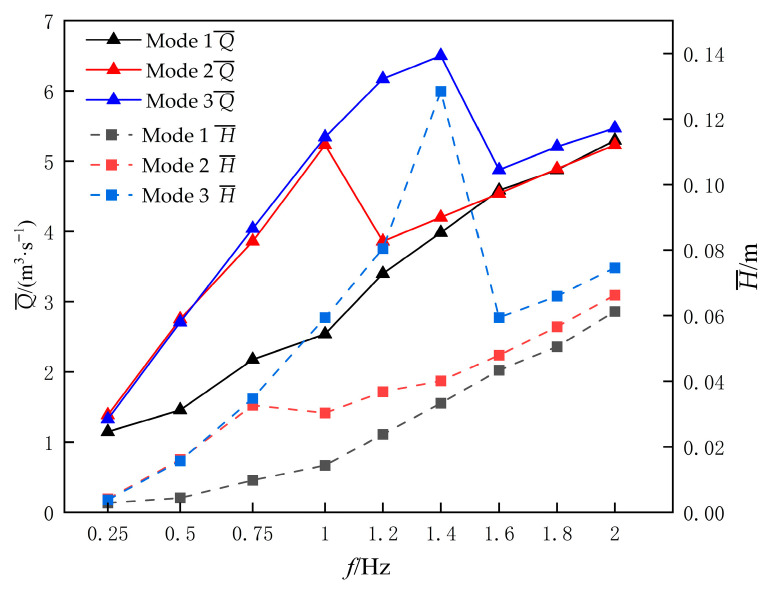
Flow rate and head profiles of flapping hydrofoils at different frequencies.

It can be observed that under low-frequency operating conditions at *f* = 0.25 Hz, the propulsion efficiencies of the three motion Modes exceed 20%. Notably, Mode 1 and Mode 2 achieve their maximum propulsion efficiencies of 23% and 24.7%, respectively, at this frequency. However, as the flapping frequency increases, the propulsion efficiencies exhibit varying degrees of decline. This phenomenon aligns with the earlier analysis: higher frequencies intensify hydrodynamic forces acting on the hydrofoil, leading to increased input power. Furthermore, due to the formation of flow recirculation zones, the growth rate of the outlet flow rate would be significantly reduced, thereby diminishing the overall propulsion efficiency. As shown in Figure 17, the variation curves of flow rate and head for the flapping hydrofoil under different frequencies are systematically compared.

As indicated in the graph, the average flow rate of Mode 1 exhibits gradual augmentation with increasing frequency. In contrast, Mode 2 demonstrates an initial increase followed by a reduction in average flow rate, subsequently resuming growth albeit with markedly diminished rate sensitivity—a phenomenon attributable to the development of flow recirculation zones. Mode 3 follows an analogous trend to Mode 2, though its flow rate degradation phase initiates at a higher critical frequency. Furthermore, all three motion Modes exhibit congruent trends between their average head variations and corresponding flow rate profiles. This correlation primarily stems from the elevated mean hydrodynamic thrust generated at higher frequencies, where intensified mechanical work imparted to the fluid medium amplifies kinetic energy transfer, thereby driving flow rate enhancement.

**Figure 18:**
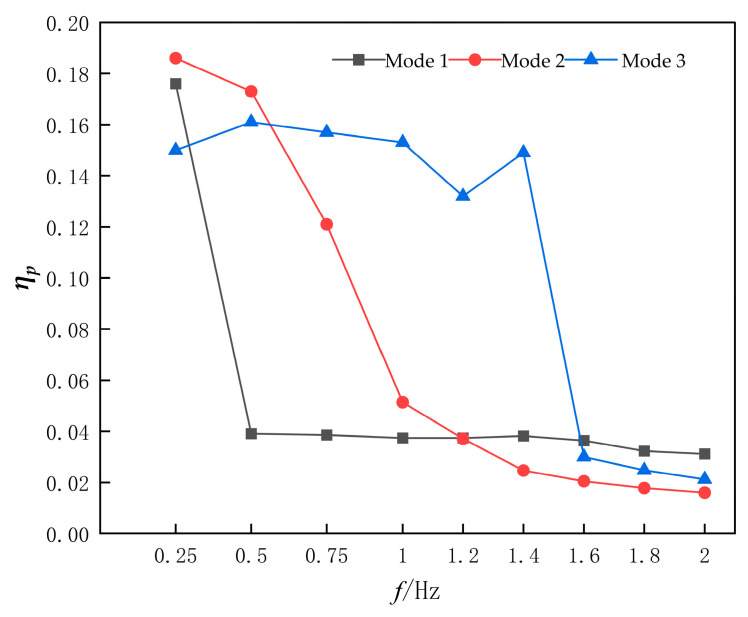
Pumping efficiency profiles of three flapping modes as a Function of Flapping Frequency.

Figure 18 presents the pumping efficiency variation curves of the device with respect to flapping frequency. The data reveal a consistent efficiency degradation across all three motion modes as frequency escalates. This observation aligns with the earlier analysis: under relatively low-frequency operating conditions (*f* < 0.75 Hz), all flapping modes are capable of generating reverse Kármán vortex streets that establish coherent jet flows for effective fluid propulsion, thereby maintaining higher pumping efficiencies. However, beyond critical frequency thresholds, efficiency deterioration initiates. Specifically, Mode 1 exhibits catastrophic efficiency collapse to 4% at *f* = 0.75 Hz due to vortex clustering near wall boundaries causing intensified energy dissipation. Mode 2 demonstrates continuous efficiency decay, reaching 5% at *f* = 1 Hz when vortex accumulation commences. Conversely, Mode 3 maintains stable pumping efficiency around 15% within 0.25 Hz ≤ *f* ≤ 1.4 Hz, but undergoes abrupt efficiency plunge to 3% at *f* = 1.6 Hz triggered by critical vorticity accumulation.

Therefore, integrating the aforementioned analyses, it can be concluded that distinct motion modes fundamentally govern vortex system formation. Comparative analysis between motion Mode 1 and Mode 2 reveals that asymmetry in heaving velocity profiles induces lateral migration of reverse Kármán vortex streets generated by hydrofoil motion, thereby inducing hydrodynamic performance degradation at elevated frequencies. Similarly, comparative evaluation of Mode 2 versus Mode 3 demonstrates that discontinuities in pitching angle modulation profoundly influence boundary-layer vortex shedding dynamics and spatiotemporal distribution methods within wake regions, which similarly drive high-frequency hydrodynamic performance deterioration. These findings exhibit strong consistency with the vortex evolution mechanisms documented in Reference [32].

### 4.4. Experimental Verification

**Figure 19:**
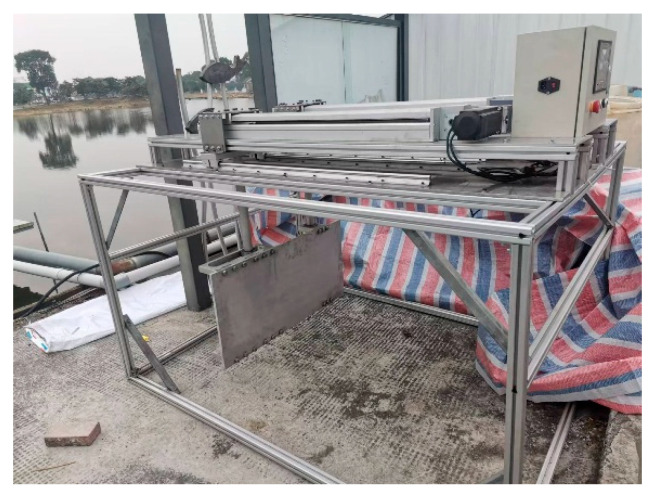
Schematic of the Synchronous Belt Mechanism.

To validate the pumping performance of the apparatus, an experimental setup for the flapping hydrofoil was designed as shown in Figure 19. The rigid flat-plate hydrofoil was tested in a 20 m × 5 m × 2 m rectangular recirculating flume, with flow velocity measurements acquired using a JD-LS6C acoustic Doppler velocimeter (Shandong Jingdao Optoelectronic Technology Co., Ltd.‌, Weifang, China). This instrument features a velocity range of 0–3 m/s, measurement accuracy of 0.005 m/s, and a maximum sampling frequency of 50 Hz.

**Figure 20:**
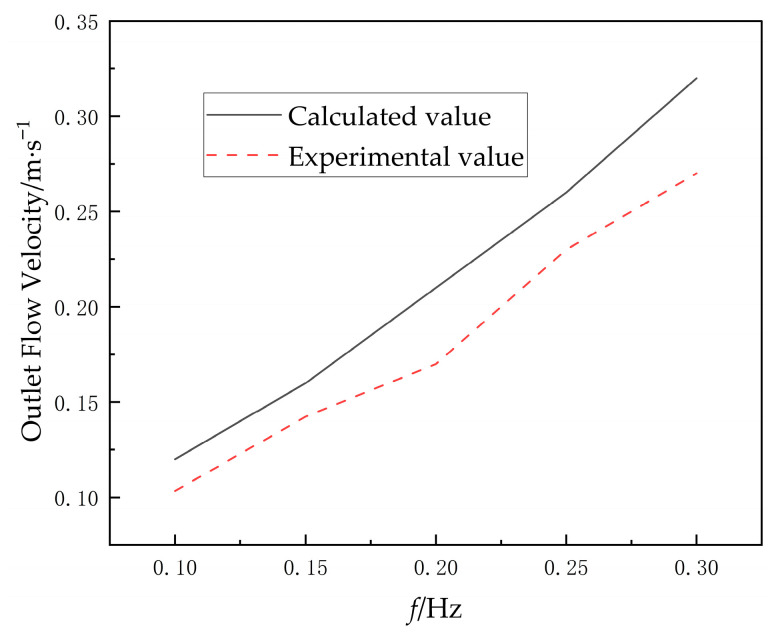
Comparison of flow velocity between test and simulation.

The flow velocity at the outlet of a square duct was measured using a current meter at 5 uniformly distributed observation points. The average velocity from these 5 measuring points served as the plane velocity. The experimental flow velocity was then compared with the simulation results, as shown in Figure 20.

The experimental data exhibit consistently higher values than the simulation results, the computational analysis yields an average relative error of 17% for the simulation results, with a 95% confidence interval of ±9% relative to experimental measurements. This discrepancy is primarily attributed to inherent differences between the experimental setup and numerical simulation conditions. Specifically, the numerical model employed a two-dimensional computational framework, which inherently cannot capture potential spanwise flow components along the hydrofoil’s length. This limitation leads to an underprediction of outlet flow velocity in simulations.

## 5. Conclusions

This study analyzes three flapping hydrofoil mechanisms designed based on sinusoidal kinematics, verifies their feasibility through dynamic simulations, and evaluates their power consumption. By establishing the hydrofoil’s motion model and employing overset mesh technology with the Realizable *k-ε* turbulence model, we systematically investigate hydrodynamic forces, flow field structures and pumping performance. Through comparative analysis, the main findings are summarized as follows:

- Due to its quick-return characteristic, the crank-slider mechanism causes the hydrofoil motion to deviate from sinusoidal kinematics. During pitch reversal, the hydrofoil experiences pressure drag from angle-of-attack stall. The cylindrical cam mechanism maintains continuous thrust despite asymmetric motion induced by profile fitting errors. In contrast, the synchronous belt mechanism strictly adheres to sinusoidal motion through precision control, achieving stable thrust throughout the entire cycle.
- At identical frequencies, the crank-slider mechanism exhibits the lowest energy consumption owing to its linkage self-locking effect and low-friction transmission. The cylindrical cam mechanism demonstrates moderate energy consumption due to contact force jumps, while the synchronous belt mechanism incurs the highest energy consumption constrained by preload losses.
- A critical frequency exists—fundamentally representing the vortex shedding desynchronization threshold—below which all mechanisms exhibit optimal hydrodynamic performance. Beyond this threshold, performance degrades significantly. The critical frequencies for the three mechanisms are 0.25 Hz, 0.75 Hz, and 1.4 Hz, respectively. The synchronous belt mechanism achieves peak propulsion efficiency of 24.3% at 1 Hz (6 times higher than the others at this frequency), leveraging its superior high-frequency adaptability. Conversely, the crank-slider and cylindrical cam mechanisms suffer drastic performance decay at high frequencies due to inertial limitations.

Therefore, in practical water-pumping applications, the crank-slider mechanism is suitable for low-frequency, small-flow-rate conditions; the cam mechanism applies to medium-frequency, moderate-flow-rate conditions; while the synchronous belt mechanism is ideal for high-frequency, large-flow-rate conditions.

At present, the experimental evidence is limited to outlet-flow-rate measurements obtained with the synchronous-belt prototype; data on instantaneous thrust, lift, or overall mechanical efficiency are not yet available because of the absence of waterproof six-axis load cells and high-speed optical diagnostics. Consequently, the energy-efficiency figures reported herein remain simulation-based. Comprehensive validation of all three mechanisms—including direct force/torque measurements and long-term reliability tests—will be undertaken as soon as the required instrumentation is commissioned.

## Data Availability

All data, models, and code generated or used during the study appear in the submitted article.

## References

[1] Zuo Q., Chen H., Dou M., Zhang Y., Li D. (2015). Experimental Analysis of the Impact of Sluice Regulation on Water Quality in the Highly Polluted Huai River Basin, China. Environ. Monit. Assess..

[2] Yuan D., Zhang Y., Liu J., Gao S., Song X. (2015). Water Quantity and Quality Joint-Operation Modeling of Dams and Floodgates in Huai River Basin, China. J. Water Resour. Plan. Manag..

[3] Jiao W.X., Cheng L., Yan H.Q., Jiang H.Y. (2019). Pressure Fluctuation Characteristics of Bidirectional Pumping System under Super-Low Head Operation. J. Hydroelectr. Eng..

[4] Zheng Y., Chen Y.J., Zhang R., Ge X.F., Lin G.P., Sun A.R. (2017). Analysis on Unsteady Stall Flow Characteristics of Axial-Flow Pump. Trans. Chin. Soc. Agric. Mach..

[5] Hua E.T., Chen W.Q., Tang S.W., Xie R.S., Guo X.M., Xu G.H. (2022). Water pushing flow characteristics of flapping hydrofoil device in small river. Trans. Chin. Soc. Agric. Mach..

[6] Zhang B., Hu X., Yang G.D., Long Z. (2021). Hydrodynamics of juvenile grass carp (*Ctenopharyngodon idellus*) in steady swimming based on pressure field. J. Hydroelectr. Eng..

[7] Chen W.S., Xia D., Liu J.K., Shi S.J. (2011). Effect of kinematic behavior of caudal fin on fishlike robot propulsion during steady swimming. J. Mech. Eng..

[8] Yang L., Su Y.M. (2011). CFD simulation of flow features and vorticity structures in tuna-like swimming. China Ocean Eng..

[9] Koochesfahani M.M. (1989). Vortical patterns in the wake of an oscillating airfoil. AIAA J..

[10] Karman T.V. (1963). General aerodynamic theory. Perfect Fluids Aerodyn. Theory.

[11] Xu J.A., Kong D.H., Gao X. (2017). Performance Analysis and Experimental Study on 2-Degree-of-Freedom Oscillating Hydrofoil Propulsion. Robot.

[12] Ding H., Song B.W., Tian W.L. (2013). Exploring Propulsion Performance Analysis of Bionic Flapping Hydrofoil. J. Northwestern Polytech. Univ..

[13] Du X.X., Zhang Z.D. (2018). Numerical Analysis of Influence of Four Flapping Modes on Propulsion Performance of Underwater Flapping Foils. Eng. Mech..

[14] Zhang S.H., Mei L., Zhou J.W. (2021). Numerical prediction of hydrodynamic performance of differently shaped flapping foil propulsors. Chin. J. Ship Res..

[15] Gu C.Y., Zhang Z.T., Xu P., Cao Y.X. (2019). Hydrodynamic Experiment and Mechanism of Improved Oscillating Hydrofoil. J. Huazhong Univ. Sci. Technol. (Nat. Sci. Ed.).

[16] Xu W., Xu G., Jiao J. (2024). Experimental Study of the Propulsive Performance and Wake Interactions of Tandem Flapping Foils. Ocean Eng..

[17] Li G.Z., Chang X., Deng N.Y., Yu P.Y. (2022). Research on the Propulsive Performance of Non-Sinusoidal Plunging In-Line Tandem Hydrofoils. Ship Sci. Technol..

[18] Chao L.M., Pan G., Zhang D., Yan G.X. (2019). Numerical Investigations on the Force Generation and Wake Structures of a Nonsinusoidal Pitching Foil. J. Fluids Struct..

[19] Chen X., Huang Q.G., Cao Y., Pan G. (2022). Nonsinusoidal Motion Effect on Self-Propelled Pitching Foil. J. Huazhong Univ. Sci. Technol. (Nat. Sci. Ed.).

[20] Li W.Z., Bao Y.D. (2023). Effects of Motion Parameters on Energy Harvesting Performance of Flapping Foils. J. Hydroelectr. Eng..

[21] Hua E., Wang T., Xiang M., Lu C., Song Y., Sun Q. (2024). Study on the Influence of Chord Length and Frequency of Hydrofoil Device on the Discharge Characteristics of Floating Matter in Raceway Aquaculture. J. Mar. Sci. Eng..

[22] Su B.Q., Wang T.M., Liang J.H., Li P. (2009). Parallel Mechanism Design on Biomimetic Tail Fin Propulsion. J. Mech. Eng..

[23] Font D., Tresanchez M., Siegenthaler C., Pallejà T., Teixidó M., Pradalier C., Palacin J. (2011). Design and Implementation of a Biomimetic Turtle Hydrofoil for an Autonomous Underwater Vehicle. Sensors.

[24] Zhang H.W., Zhang J.C., Wang Y.H., Ma B. (2017). Analysis on Hydrodynamic Characteristics of Asymmetric-Flapping Foil and the Mechanism Design. J. Mach. Des..

[25] Hu R., Wang W.B., Guo Q.L., Wang H.T., Cao F. (2010). Design of Bionic Robofish with Flapping Wing. J. Shihezi Univ. (Nat. Sci.).

[26] Niu C., Zhang L., Bi S., Cai Y. Mechanical Design and Implementation of a Bio-Inspired Robotic Fish with Flapping Foils. Proceedings of the 2013 IEEE International Conference on Robotics and Biomimetics (ROBIO).

[27] Gao J., Bi S.S., Li J., Cai Y.R. (2011). Design and Hydrodynamic Experiments on Robotic Fish with Oscillating Pectoral Fins. J. Beijing Univ. Aeronaut. Astronaut..

[28] Hua E.T., Su Z.X., Xie R.S., Chen W.Q., Tang S.W., Luo H.T. (2023). Optimization and Experimental Verification of Pivot Position of Flapping Hydrofoil. J. Hydroelectr. Eng..

[29] Hua E., Zhu W., Xie R., Su Z., Luo H., Qiu L. (2023). Comparative Analysis of the Hydrodynamic Performance of Arc and Linear Flapping Hydrofoils. Processes.

[30] Launder B.E., Spalding D.B. (1974). The Numerical Computation of Turbulent Flows. Comput. Methods Appl. Mech. Eng..

[31] Read D.A., Hover F.S., Triantafyllou M.S. (2003). Forces on Oscillating Foils for Propulsion and Maneuvering. J. Fluids Struct..

[32] Liu H.X., Su Y.M., Pang Y.J. (2016). Effects of the Nonsinusoidal Oscillating on the Hydrodynamic Performance of the Hydrofoil. J. Huazhong Univ. Sci. Technol. (Nat. Sci. Ed.).

